# Characterization of bioactive substances involved in the induction of bone augmentation using demineralized bone sheets

**DOI:** 10.1186/s40729-022-00449-9

**Published:** 2022-11-01

**Authors:** Haruka Saito, Risako Chiba-Ohkuma, Yasuo Yamakoshi, Takeo Karakida, Ryuji Yamamoto, Mai Shirai, Chikahiro Ohkubo

**Affiliations:** 1 Department of Removable Prosthodontics, School of Dental Medicine, Tsurumi University, 2-1-3 Tsurumi, Tsurumi-Ku, Yokohama, 230-8501 Japan; 2 Department of Biochemistry and Molecular Biology, School of Dental Medicine, Tsurumi University, 2-1-3 Tsurumi, Tsurumi-Ku, Yokohama, 230-8501 Japan

**Keywords:** Guided bone regeneration, Ridge preservation, Resorbable membrane, Bone augmentation, Transforming growth factor-beta, Dentin matrix protein 1, Matrix extracellular phosphoglycoprotein, Biglycan

## Abstract

**Purpose:**

To investigate the bone augmentation ability of demineralized bone sheets mixed with allogeneic bone with protein fractions containing bioactive substances and the interaction between coexisting bioactive substances and proteins.

**Methods:**

Four types of demineralized bone sheets mixed with allogeneic bone in the presence or absence of bone proteins were created. Transplantation experiments using each demineralized bone sheet were performed in rats, and their ability to induce bone augmentation was analysed by microcomputed tomography images. Bioactive substances in bone proteins were isolated by heparin affinity chromatography and detected by the measurement of alkaline phosphatase activity in human periodontal ligament cells and dual luciferase assays. Noncollagenous proteins (NCPs) coexisting with the bioactive substances were identified by mass spectrometry, and their interaction with bioactive substances was investigated by in vitro binding experiments.

**Results:**

Demineralized bone sheets containing bone proteins possessed the ability to induce bone augmentation. Bone proteins were isolated into five fractions by heparin affinity chromatography, and transforming growth factor-beta (TGF-β) was detected in the third fraction (Hep-c). Dentin matrix protein 1 (DMP1), matrix extracellular phosphoglycoprotein (MEPE), and biglycan (BGN) also coexisted in Hep-c, and the binding of these proteins to TGF-β increased TGF-β activity by approximately 14.7% to 32.7%.

**Conclusions:**

Demineralized bone sheets are capable of inducing bone augmentation, and this ability is mainly due to TGF-β in the bone protein mixed with the sheets. The activity of TGF-β is maintained when binding to bone NCPs such as DMP1, MEPE, and BGN in the sheets.

## Background

After a dental defect, alveolar bone resorption occurs, and it deceptively appears as a morphological change of the alveolar ridge. This phenomenon is related to various factors, such as the cause of tooth extraction, the time elapsed after tooth extraction, the occlusal relationship, denture wearing experience, denture use, denture structure and materials, age, sex, and metabolic regulation mechanisms of hormones and vitamins [1, 2]. Therefore, the appearance of the disease is not always uniform and varies from one individual to another. In the prosthetic area, the morphological change of the alveolar bone is an important issue related to the availability, aesthetics, and longevity of implant treatment, and it is necessary to consider reconstruction for hard and soft tissues while understanding the site and direction of alveolar bone resorption and their changes over time.

Implant treatments such as guided bone regeneration (GBR), sinus lifting, socket lifting, and ridge preservation that aim to increase bone mass after tooth extraction utilize autogenous bone graft material, or bone replacement materials classified as allogeneic bone, heterogeneous bone and synthetic artificial bone. Several studies on the use of ridge preservation to minimize postextraction bone resorption have shown that this technique is effective in reducing horizontal and vertical bone resorption after tooth extraction [3–5]. In GBR and ridge preservation, nonresorbable membranes, such as expanded polytetrafluoroethylene and titanium mesh, an resorbable membranes, such as synthetic polymeric and lactic/glycolic acid, have been used, but no significant differences in barrier effects on postoperative infection, wound healing, or exposure of the membrane or grafting material has been observed [6]. In recent years, some studies have been conducted on the use of absorbable collagen membranes and absorbable collagen sponges to inhibit bone resorption and improve bone augmentation [4, 7, 8]. Furthermore, the combination of lyophilized bone allografts with platelet-rich fibrin or growth factor-rich plasma has been shown to contribute for the efficacy in alveolar ridge preservation [9, 10]. In our previous study, we developed a novel sheet of demineralized rat femur bone (demineralized bone sheet) and demonstrated that bone augmentation was clearly promoted by covering the demineralized bone sheet at the location of the implant immediately after placement into the extraction socket [11].

Bioactive substances such as bone morphogenetic proteins (BMP), transforming growth factor-beta (TGF-β), fibroblast growth factor, and insulin-like growth factor (IGF) are present in the bone matrix [12–16] and play an important role in bone remodelling while participating in the induction of osteoblast chemotaxis, proliferation, and differentiation. Indeed, bioactive osteogenic substances in bone extracts have been shown to induce ectopic bone formation and stimulate bone marrow stromal cells to differentiate into bone and cartilage tissue [17]. We hypothesized that bioactive substances contained in demineralized bone sheets must interact with proteins exist in the same environment to exert their functions.

Here, we report the bone augmentation ability of demineralized bone sheets mixed with allogeneic bone with protein fractions containing bioactive substances and the interaction between the coexisting bioactive substances and proteins.

## Materials and methods

### Preparation of demineralized bone sheets

Femora were harvested from 6- to 10-week-old male Sprague–Dawley (SD) rats and used to prepare demineralized bone sheets. Following the removal of bone marrow from the collected bone, spongy bone and periosteum were mechanically removed and rinsed with phosphate-buffered saline (PBS) using an ultrasonic cleaner. Femora were washed twice with 50 mM Tris–HCl/4 M guanidine buffer (pH 7.4) containing cOmplete™ ULTRA tablets, EDTA-free (Roche, Basle, Switzerland), and 1 mM 1,10-phenanthroline (Sigma-Aldrich, St. Louis, MO, USA) (TG buffer) for 2 h at 4 °C. The TG-soluble fraction was removed, and the remaining femora were demineralized with 0.5 M HCl containing the above inhibitors for 48 h at 4 °C, followed by the removal of the HCl-soluble fraction. The demineralized femora were then trimmed to generate fragments of approximately 3.0 mm (width) × 3.0 mm (depth) × 0.3 mm (height) (DBS-P) with a digital micrometer (range of measurement: 0–25 mm, minimum display amount: 0.001 mm) (Mitutoyo, Kawasaki, Japan). Some of the DBS-P samples were further treated with TG buffer for 12 h at 4 °C, and the soluble fraction (the G2 extract) was dialyzed against water for 3 days, with the water changed every day, and lyophilized. This demineralized bone sheet, which did not contain the G2 extract, was designated DBS-E. Some of the DBS-E samples were remixed with the G2 extract in a dialysis tube and treated with PBS for 4 days at 4 °C. This demineralized bone sheet was designated DBS-R. Separately, the DBS-E sample was suspended with PBS only and treated in the same way without the G2 extract and was designated DBS-C.

### Animal transplantation experiment and quantitative computed tomography

Four types of demineralized bone sheets (DBS-P, DBS-E, DBS-R and DBS-C) were transplanted subcutaneously under the axilla of 6-week-old male SD rats (*n* = 6). The rats were sacrificed 8 weeks later, and the demineralized bone sheets were recovered. Each demineralized bone sheet was photographed with a microcomputed tomography (micro-CT) system (inspeXio SMX-225CT, Shimadzu, Kyoto, Japan). The imaging conditions were a tube voltage of 115 kV, a tube current of 70 μA, a resolution of 1024 × 1024 pixels, operating conditions of 1200 views, and a total scanning time of 900 s. Bone mineral density (BMD) images were generated by taking known phantoms under the same conditions as a demineralized bone sheet and taking BMD values as pixel values from micro-CT images. The obtained micro-CT image was image-analyzed with three-dimensional reconstruction software (TRI/3D-BON-FCS64, Ratoc, Tokyo, Japan).

### Preparation of bone powder and extraction of bone proteins

The tibiae and femora were collected from 6-week-old SD rats and pulverized to a powder using a jaw crusher (Retsch, Newtown, PA, USA). Then, 20 g of bone powder was homogenized at 4 °C in 400 mL of TG buffer. The insoluble material was pelleted by centrifugation (15,900×*g*) and homogenized twice more in guanidine buffer. The soluble fraction (G1 extract) was combined and stored at − 80 °C. The insoluble pellet was placed in a Spectra/Por 3 dialysis tube (MW = 3500 cut off) (Spectrum Laboratories Inc., Rancho Dominguez, CA, USA) and demineralized against 4 L of 0.1 M HCl. After 5 days, the dialysis tube contents were centrifuged, and the supernatant was designated the acid extract (H extract). The insoluble pellet was homogenized at 4 °C in 400 mL of TG buffer. The insoluble material was pelleted by centrifugation (15,900×*g*) and homogenized twice more in TG buffer. The supernatants were combined and designated the second guanidine extract (the G2 extract). The insoluble pellet (RIM: residue insoluble materials) was rinsed with water and lyophilized. The G1, H and G2 extracts were dialyzed against water for 3 days, with the water changed every day, and lyophilized. Each extract was stored at − 80 °C. Aliquots of the G2 extract (250 μg/mL) were used for the alkaline phosphatase (ALP) activity assay and dual-luciferase reporter gene assay.

### Alkaline phosphatase (ALP) activity assay

Human periodontal ligament fibroblasts (HPDL cells) were obtained from LONZA (Lonza, Walkersville, MD, USA). The HPDL cells were distributed in 96-well plates at a density of 1.0 × 10^4^ cells/well and incubated at 37 °C for 24 h in a humidified atmosphere with 5% CO_2_ using a standard medium consisting of alpha minimum essential medium (#12571-063, Thermo Fisher Scientific, Waltham, MA, USA) containing 10% foetal bovine serum (Cosmo Bio, Tokyo, Japan; CCP-FBS-BR-500) and 1% penicillin–streptomycin (5000 U/mL) (#15070-63, Thermo Fisher Scientific). The medium was changed to growth medium supplemented with 10 μg/mL samples with or without 1 μM SB43152 (SB) (ChemScence, Monmouth Junction, NJ, USA). The process used to measure ALP activity in each well was described previously [18]. After 72 additional hours of incubation, the cells were washed once with PBS, and ALP activity was assayed using 10 mM *p*-nitrophenylphosphate as the substrate in 100 mM 2-amino-2-methyl-1,3-propanediol-HCl buffer (pH 10.0) containing 5 mM MgCl_2_, with incubation for 10 min at 37 °C. Sodium hydroxide (0.2 M) was added to quench the reaction, and the absorbance at 405 nm was read on a plate reader.

### Heparin affinity chromatography

Fifty milligrams of the G2 extract were dissolved in 50 mM Tris–HCl/6 M urea buffer (pH 7.4) and separated by affinity chromatography using a Heparin Sepharose 6 Fast Flow column (1.6 cm × 20 cm, Cytiva, Marlborough, MA, USA) equilibrated with buffer A: 50 mM Tris–HCl and 6 M urea (pH 7.4). Proteins were eluted with buffer A and a linear gradient of buffer B (buffer A + 0.05 M NaCl), C (buffer A + 0.1 M NaCl), D (buffer A + 0.2 M NaCl) and E (buffer A + 1 M NaCl) for 5 h at a flow rate of 0.2 mL/min at 4 °C. Fractions were collected every 20 min, and the absorbance at 280 nm was determined. Five fractions were dialyzed against water for 3 days, with the water changed every day, and lyophilized. Each fraction was stored at − 80 °C. Aliquots of samples (5 μg/mL) were used for the ALP activity assay.

### SDS–polyacrylamide gel electrophoresis (SDS–PAGE)

SDS–PAGE was performed using 5–20% e-PAGEL minigels (ATTO Corporation, Tokyo, Japan). Samples (10 μg) were dissolved in NuPAGE® LDS sample buffer (Invitrogen, Carlsbad, CA, USA), and electrophoresis was carried out using a current of 30 mA for 1 h. The gels were stained with Simply Blue™ Safe Stain (Invitrogen), Stains-all (Sigma, St. Louis, MO, USA), and ProteoSilver™ Silver Stain Kit (Sigma). The apparent molecular weights of the protein bands were estimated by comparison with the SeeBlue® Plus2 Pre-Stained Standard (Invitrogen).

### Dual-luciferase reporter-gene assay for TGF-β activity

An enamel epithelial cell line (mHAT9d), which was derived from the apex of a mouse tooth, was seeded into 96-well plates at a density of 1.5 × 10^4^ cells/well and then cultured with a standard medium consisting of Dulbecco's modified Eagle’s medium: nutrient mixture F-12 (DMEM/F-12) (#11320-033, Thermo Fisher Scientific) containing 10% foetal bovine serum (Cosmo Bio) and 1% penicillin–streptomycin (5000 U/mL) (Thermo Fisher Scientific). Both p3TP-lux (Addgene, Cambridge, MA, USA), conjugated to the TGF-β-responsive plasminogen activator inhibitor-1 (*PAI-1*) promoter region, and pRL-SV 40 (Promega Co., Madison, WI, USA), an internal standard, were simultaneously transfected into mHAT9d cells using Lipofectamine 3000 (Invitrogen). The G2 extract (250 μg/mL) was added, and the cells were cultured for 24 h. Luciferase reporter-gene assays were performed according to the manufacturer’s instructions (Dual-Luciferase Reporter Assay System, Promega), and luciferase activity was measured using Minilumat Lb 9506 (Berthold GmbH & Co., Bad Wildbad, Germany).

### Data-independent acquisition mass spectrometry (DIA-MS)

DIA-MS (Kazusa DNA Res. Inst., Chiba, Japan) was performed using 50 μg of Hep-c. The identification and quantitative results were analysed with Scaffold DIA software, and proteins that had both a peptide false discovery rate (FDR) < 1% and a protein FDR < 1% were selected.

### In vitro binding experiments

Carrier-free (CF)-recombinant human dentin matrix protein 1 (CF-hDMP1) (#4129-DM-050, R&D Systems, Minneapolis, MN, USA), CF-recombinant human matrix extracellular phosphoglycoprotein (CF-hMEPE) (#3140-ME-050, R&D Systems) and CF-recombinant human biglycan (CF-hBGN) (#2667-CM-050, R&D Systems) (20 μg each) were incubated with 1 µg of CF-human recombinant TGF-β1 (CF-hTGF-β1) (#8915, Cell Signaling Technology, Danvers, MA, USA) in 50 mM Tris–HCl buffer (pH 7.4) for 20 h at 37 °C. Each sample was fractionated by ion exchange-high-performance liquid chromatography (IE-HPLC) in an Inertsil AX column (0.46 × 25 cm; GL Sciences Inc., Tokyo, Japan) run at a flow rate of 0.5 mL/min and monitored at 220 nm [buffer A, 50 mM Tris–HCl/6 M urea (pH 7.4); buffer B, 1 M NaCl/buffer A]. Proteins were eluted with a linear gradient of buffer B for 55 min at a flow rate of 0.5 mL/min, and 1-mL fractions were collected. Each fraction was desalted and buffer-exchanged to 50 mM Tris–HCl buffer (pH 7.4) in an Amicon Ultra-3K (Merck KGaA, Darmstadt, Germany). Each fraction was concentrated to a 50 µL volume, and aliquots (10 µL) were used for the ALP activity assay. CF-hDMP1, CF-hMEPE, CF-hBGN, and CF-hTGF-β1 only were incubated and fractionated by IE-HPLC as controls.

### Statistical analysis

All values of ALP activity and luciferase assays are presented as a box-and-whisker plot. Statistical significance was determined using the Mann–Whitney *U* test and *p* < 0.05 was considered statistically significant.

## Results

### Preparation of demineralized bone sheets and animal transplantation experiments

**Fig. 1:**
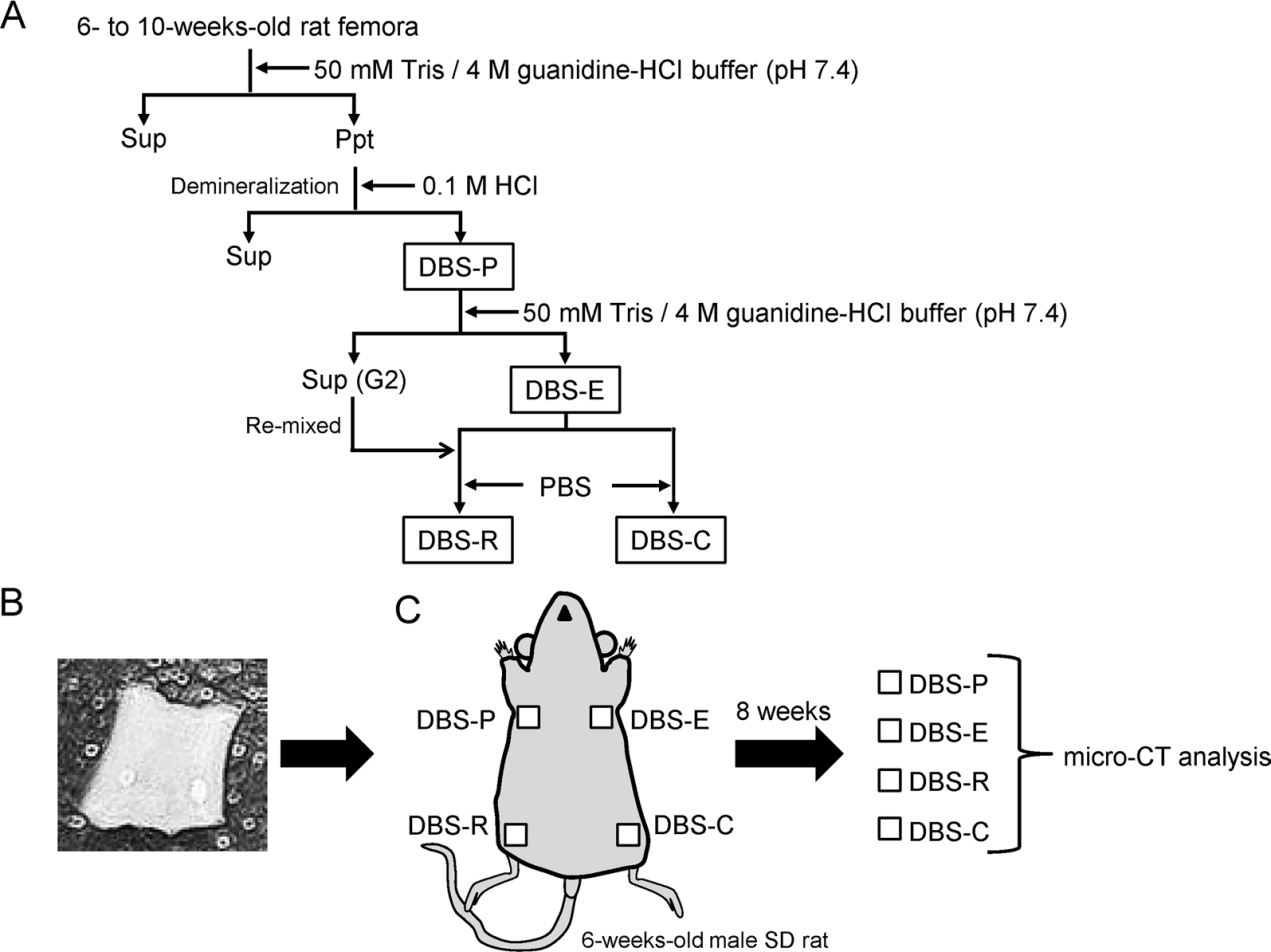
Preparation of demineralized bone sheets and animal transplantation experiments. **A** Flow chart showing the procedures used to generate four types of demineralized bone sheets. Sup: supernatant, Ppt: precipitate. **B** Photograph of a representative demineralized bone sheet. Each demineralized bone sheet was trimmed to approximately 3.0 mm (width) × 3.0 mm (depth) × 0.3 mm (height). **C** Schematic diagram showing the procedures for the animal transplantation experiment. The demineralized bone sheets, DBS-P, DBS-E, DBS-R and DBS-C, were transplanted subcutaneously under the axilla of 6-week-old male SD rats. The rats were sacrificed 8 weeks later, and the demineralized bone sheets were recovered. Each demineralized bone sheet was analysed with a micro-CT system

To generate demineralized bone sheets, femora were subjected to a series of three extractions: a first guanidine extraction, HCl demineralization, and a second guanidine extraction (Fig. 1A). We trimmed the femora after demineralization with HCl to approximately 3.0 mm (width) × 3.0 mm (depth) × 0.3 mm (height) fragments with a digital micrometer (Fig. 1B). Using this demineralized bone sheet (DBS-P), we made three more sheets (DBS-E, DBS-R and DBS-C) according to the protocol. We transplanted four types of demineralized bone sheets subcutaneously under the axilla of rats according to the protocol illustrated in Fig. 1C, followed by micro-CT analysis. No post-transplant rejection was observed in this experiment.

**Fig. 2:**
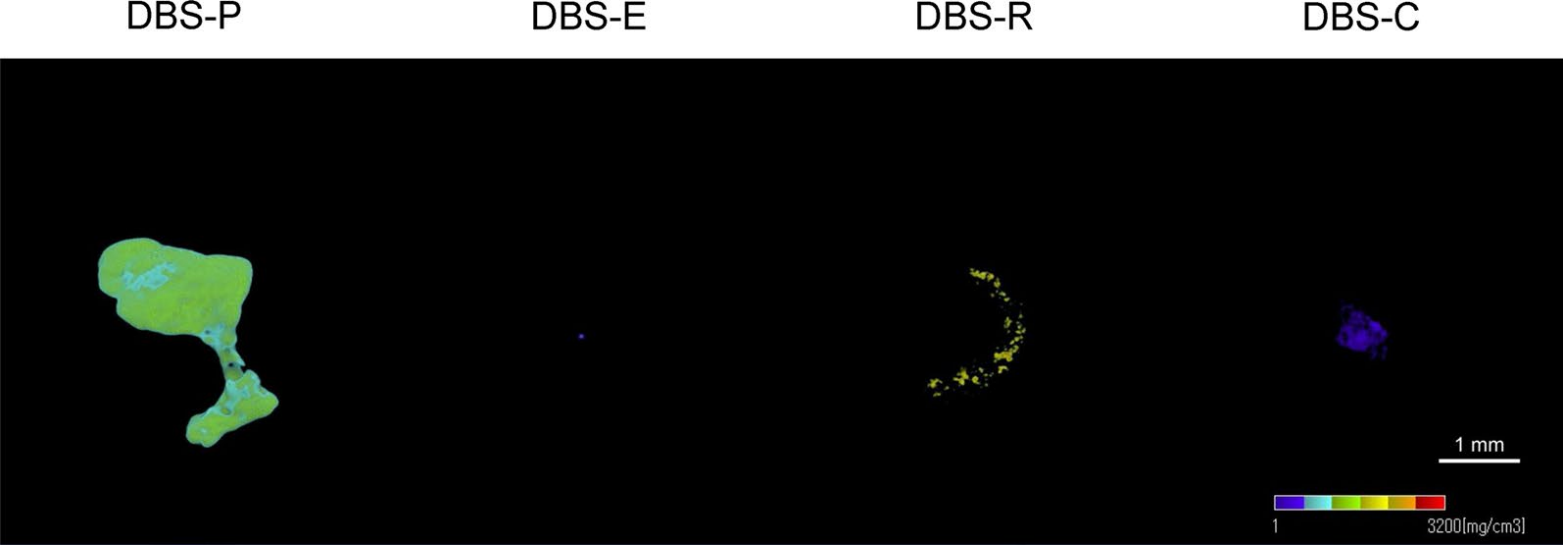
Bone density imaging by quantitative computed tomography analysis of four types of demineralized bone sheet 8 weeks after transplantation. Imaging conditions: tube voltage, 115 kV; tube current, 70 μA; resolution, 1024 × 1024 pixels; operating conditions, 1200 views; and total scanning time, 900 s. BMD images were generated by taking known phantoms under the same conditions as the demineralized bone sheets and taking the BMD values as pixel values from micro-CT images. The obtained micro-CT image was image-analyzed with three-dimensional reconstruction software

Figure 2 shows bone density imaging by quantitative computed tomography analysis of each demineralized bone sheet 8 weeks after transplantation. Of the bone densities assessed by imaging with bone mineral quantitation phantoms, DBS-P showed the widest bone density distribution (approximately 530–1060 mg/cm^3^). DBS-R was interspersed with a higher bone density region (approximately 1600 mg/cm^3^) than DBS-P. In contrast, the bone density was rarely measured for DBS-E and DBS-C.

### Sequential extraction of bone proteins

**Fig. 3:**
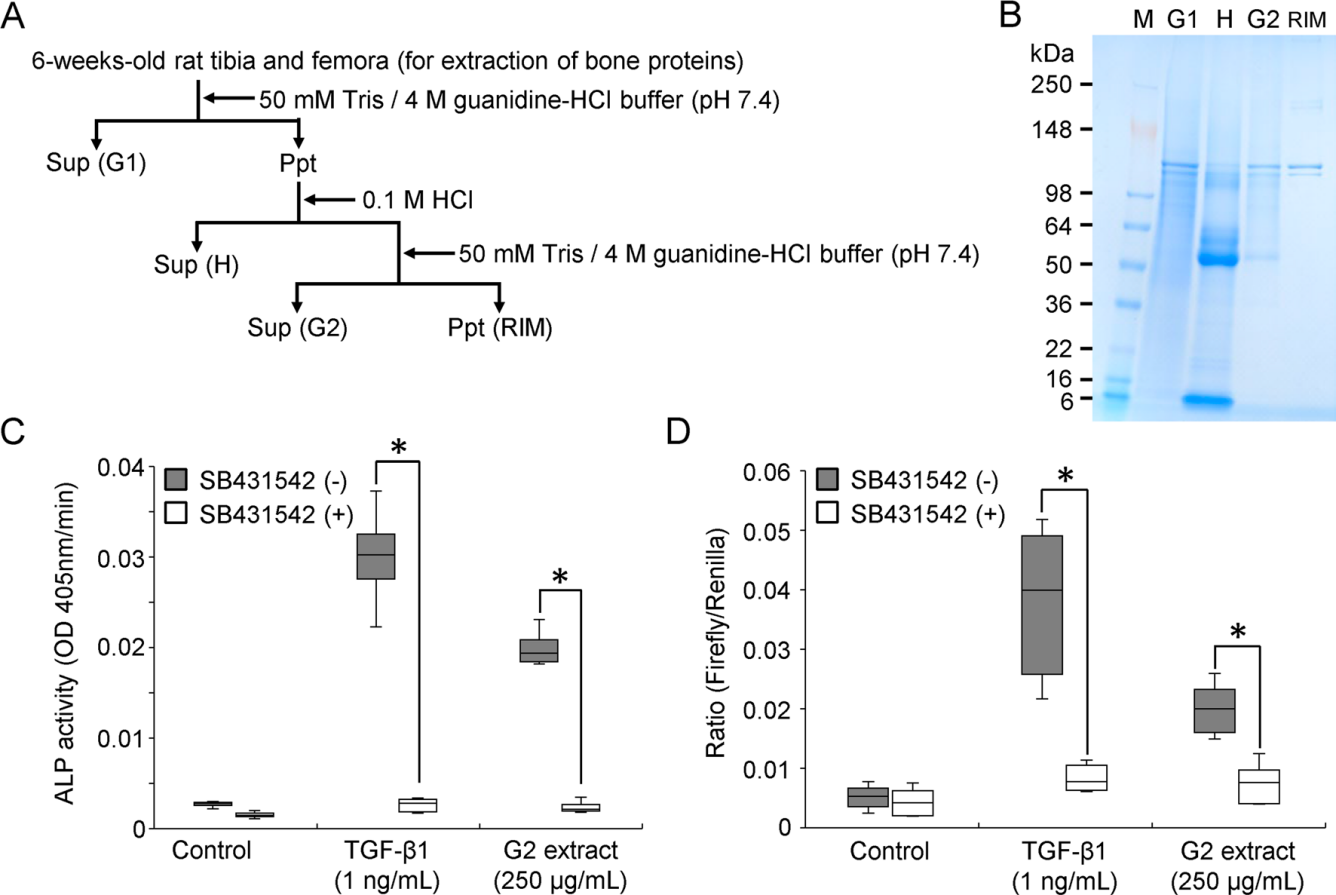
Detection of TGF-β activity in the G2 extract. **A** Flow chart showing the procedures used for the primary extracts of proteins from bone powder. Sup: supernatant, Ppt: precipitate, RIM: residual insoluble materials. **B** Rat bone powder extracts analysed by SDS–PAGE stained with Bio-Safe Coomassie Brilliant Blue. M: SeeBlue® Plus2 Pre-Stained Standard. **C** ALP-inducing activity of HPDL cells exposed to the G2 extract (250 μg/mL) and CF-hTGF-β1 (1 ng/mL) without (−) or with (+) SB431542. Data in box indicate the 25–75% tiles of the interquartile range (IQR), and the end of whiskers indicates maximum and minimum values, respectively. The median is represented as line located in the middle of box (*p < 0.05, Mann–Whitney *U* test). Control: ALP-inducing activity of HPDL cells cultured in the absence of the G2 extract or CF-hTGF-β1. **D** Dual-luciferase reporter-gene assay for the G2 extract. Firefly luciferase activity exposed to the G2 extract (250 μg/mL) and CF-hTGF-β1 (1 ng/mL) without (−) or with (+) SB431542. Firefly luciferase activity was standardized based on Renilla luciferase activity, which is an internal standard. Data in box indicate the 25–75% tiles of IQR, and the end of whiskers indicates maximum and minimum values, respectively. The median is represented as line located in the middle of box (*p < 0.05, Mann–Whitney *U* test). Control: dual-luciferase reporter gene assay of mHAT9d cells cultured in the absence of the G2 extract or CF-hTGF-β1

Since the transplantation experiment with the demineralized bone sheets revealed their ability to induce augmentation in DBS-P and DBS-R containing G2 extract, we next attempted to detect bioactive substances contained in the G2 extract. We pulverized both the tibiae and femora to a powder and sequentially extracted three protein fractions with TG buffer and HCl (Fig. 3A). These extractions yielded four fractions, the G1, H, and G2 extracts and the residual insoluble material (RIM), which were analysed by SDS–PAGE stained with Bio-Safe Coomassie Brilliant Blue (Fig. 3B). The G2 extract clearly enhanced ALP-inducing activity in HPDL cells, and the activity level in the presence of the TGF-β type I receptor kinase inhibitor SB431542 was significantly reduced (Fig. 3C). To better identify TGF-β activity, we performed a luciferase reporter gene assay. Figure 3D shows the result of the standardization of firefly luciferase activity to Renilla luciferase as an internal control. The G2 extract enhanced *PAI-1* gene promoter activity, and this activity was significantly suppressed in the presence of SB431542.

### Detection of TGF-β activity in the G2 extract

**Fig. 4:**
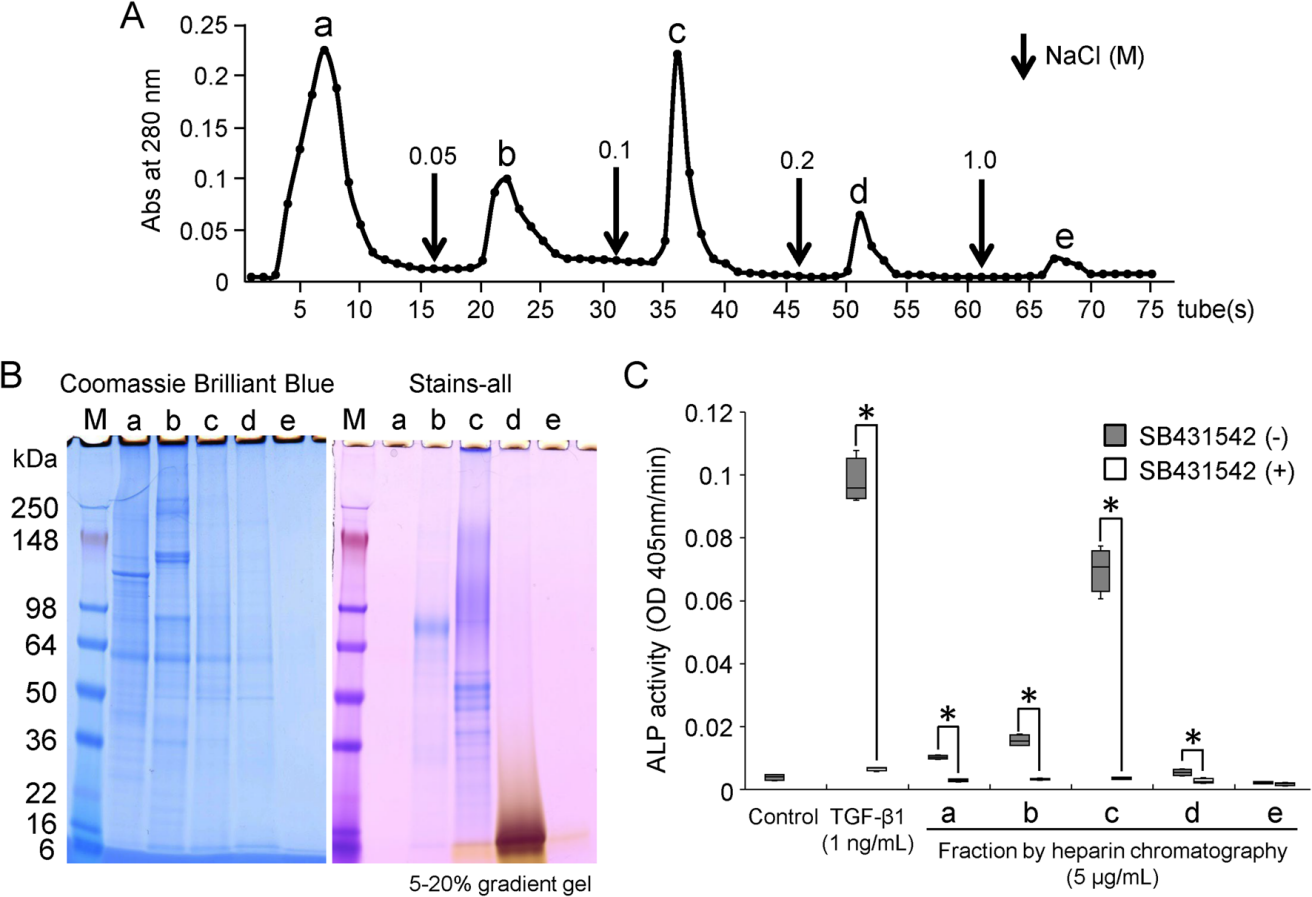
Isolation of NCPs coexisting with TGF-β in the G2 extract. **A** Heparin affinity chromatogram showing absorbance at 280 nm for the G2 extract obtained from bone powder of rat tibiae and femora (50 mg). Downward-pointing arrows are the starting point of the step gradient with 0.05, 0.1, 0.2 and 1 M NaCl. **B** SDS–PAGE (5–20% gradient gel) stained with Bio-Safe Coomassie Brilliant Blue (Coomassie Brilliant Blue, left) and Stains-all (right) showing fractions *a*, *b*, *c*, *d*, and *e* on a heparin affinity chromatogram. **C** ALP-inducing activity of HPDL cells exposed to fractions *a*, *b*, *c*, *d*, and *e* (5 μg/mL each) and CF-hTGF-β1 (1 ng/mL) without (−) or with (+) SB431542. Data in box indicate the 25-75% tiles of IQR, and the end of whiskers indicates maximum and minimum values, respectively. The median is represented as line located in the middle of box (*p < 0.05, Mann–Whitney *U* test). Control: ALP-inducing activity of HPDL cells cultured in the absence of fractions a, b, c, d, and e or CF-hTGF-β1

We further attempted to obtain information about the interaction between TGF-β and NCPs in the G2 extract. The G2 extract was separated into five fractions by heparin affinity chromatography (Fig. 4A). On SDS–PAGE, collagen α chains were predominantly observed in the first and second fractions (Hep-a and Hep-b), while NCPs with a molecular weight of approximately 25–150 kDa stained blue or purple with Stains-all staining were observed in Hep-b and the third fraction (Hep-c), respectively (Fig. 4B). Interestingly, a smeared band of NCPs that stained yellow with Stains-all staining and had a molecular weight of approximately 15–30 kDa was detected in the fourth fraction (Hep-d). Of the five fractions, Hep-c enhanced ALP-inducing activity in HPDL cells, and this activity was significantly reduced in the presence of SB431542 (Fig. 4C).

### Identification of noncollagenous proteins in Hep-c

We next identified NCPs in Hep-c by DIA–MS. Table 1 shows the identification and quantitative results obtained from Scaffold DIA after DIA–MS analysis. The DIA–MS results showed that the NCP bands corresponding to molecular weights of 25–150 kDa in Hep-c isolated by SDS–PAGE may be BGN, MEPE, and DMP1 in order of increasing quantitative values.

**Table 1:** Identification and quantitative results obtained from Scaffold DIA after DIA–MS analysis

Accession number	Protein name	Gene symbol	Molecular weight ( kDa)	Quantitative value
P47853	Biglycan	Bgn	42	2.7E+10
Q9ES02	Matrix extracellular phosphoglycoprotein	Mepe	47	2.7E+10
P98193	Dentin matrix acidic phosphoprotein 1	Dmp1	53	2.1E+10
Q01129	Decorin	Dcn	40	1.3E+10
P02770	Albumin	Alb	69	1.1E+10
P07897	Aggrecan core protein	Acan	221	3.5E+09
D3ZAF5	Periostin	Postn	90	3.3E+09

Detected proteins are listed in order of increasing quantification value

### In vitro binding experiments

**Fig. 5:**
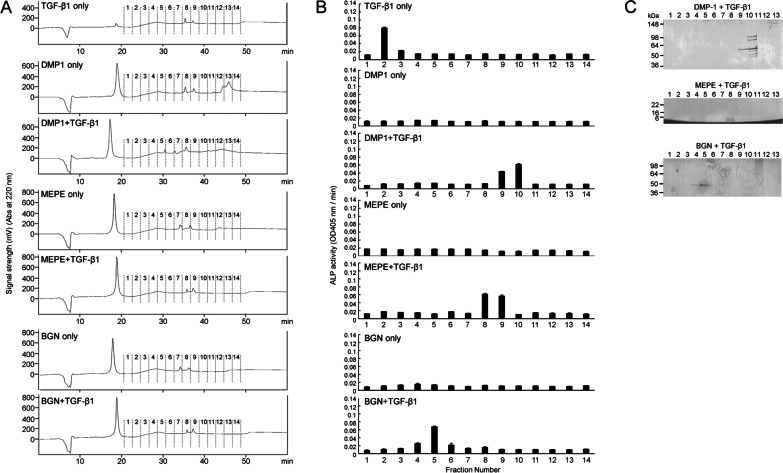
In vitro binding study of CF-hTGF-β1 with CF-hDMP1, CF-hMEPE, and CF-hBGN. **A** IE-HPLC chromatograms showing absorbance at 220 nm. **B** ALP-inducing activity of HPDL cells exposed to fractions 1 to 13 in each sample. CF-hTGF-β1 without incubation for the binding experiment (1 ng/mL) was used as a positive control. Data are means ± SEs of 3 culture wells (*p < 0.05, Mann–Whitney *U* test). **C** SDS–PAGE (5–20% gradient gel) stained with silver showing CF-hDMP1 (top), CF-hMEPE (middle), and CF-hBGN (bottom) after binding to CF-hTGF-β1 in fractions 1–13

To explore the interactions between TGF-β and the above three NCPs detected by DIA–MS, we performed in vitro binding experiments. Figure 5A and B display IE-HPLC chromatograms from the in vitro binding experiment of CF-hTGF-β1 and CF-hDMP1, CF-hMEPE, and CF-hBGN and the ALP-inducing activity of each fraction in HPDL cells. When CF-hTGF-β1 alone was incubated without recombinant NCP, the second fraction eluted by IE-HPLC enhanced ALP-inducing activity in HPDL cells. Following CF-hTGF-β1 binding, three NCPs eluted into fractions 9 and 10 for CF-hDMP1, fractions 8 and 9 for CF-hMEPE, and fractions 4 and 5 for CF-hBGN enhanced ALP-inducing activity in HPDL cells. In addition, the protein bands on SDS–PAGE for the three NCPs correlated with the positions of ALP-inducing activity (Fig. 5C). The total amounts (ng) of bound TGF-β1 in fractions obtained from IE-HPLC after binding experiments with twenty μg of CF-hDMP1, CF-hMEPE, and CF-hBGN were calculated from the standard CF-hTGF-β1 (1 ng/mL) and are shown in Table 2. When CF-hTGF-β1 alone was incubated without binding NCPs, the activity obtained from IE-HPLC was retained, but the amount after dilution correction was 112.9 ± 0.38 ng. Three NCPs were able to bind CF-hTGF-β1, and their amounts after dilution correction were 149.5 ± 0.41, 167.9 ± 0.77 and 132.4 ± 0.48 ng/20 μg for CF-hDMP1, CF-hMEPE and CF-hBGN, respectively.

**Table 2:** Amount of unbound or bound TGF-β1 for recombinant human DMP1, MEPE, and biglycan

Binding protein	Unbound (ng)	Bound (ng)
TGF-β1 only	112.9 ± 0.38	–
DMP1	–	149.5 ± 0.41
MEPE	–	167.9 ± 0.77
BGN	–	132.4 ± 0.48

The value indicates total ng of TGF-β1 obtained from IE-HPLC after binding experiments against 20 μg of protein

## Discussion

In clinical dentistry, a reduction in bone height due to alveolar bone resorption accompanying tooth extraction is inevitable, and this is an obstacle for implant treatment, which requires a constant bone height and width. Therefore, it is desired to develop a material that not only suppresses alveolar bone resorption but also achieves an ideal alveolar bone height. To date, the effectiveness of shielding membranes in bone augmentation has been reported, and various membranes are commercially available [4, 6–8]. In the present study, we created four types of demineralized bone sheets (DBS-P, DBS-E, DBS-R and DBS-C) mixed with allogeneic bone in the presence or absence of guanidine extract (i.e., G2 extract). Moreover, our animal transplantation experiment demonstrated that both DBS-P and DBS-R containing G2 extract possess the ability to induce bone augmentation. This finding indicates that the proteins and/or bioactive substances in the G2 extract are necessary to endow the demineralized bone sheet with mineralization-inducing ability. Considering that no transplantation immunity was observed in animal experiment, our result may suggest the possibility that demineralized bone sheets made of allogeneic bone can be contributed in some way for future implant treatment as bone replacement material.

TGF-β, BMP, and IGF are stored in high concentrations in the bone matrix and play important roles for the differentiation and functional regulation on of osteoblasts. Among these, TGF-β which accumulated in the bone matrix as the inactive latent form is activated, when it is released out of the bone matrix by bone resorption. Subsequently, the active TGF-β promotes the chemotaxis of osteoblasts to sites of bone resorption, and the production of bone matrix proteins [19, 20]. In this way, TGF-β has been extensively studied as a coupling factor in bone remodelling. In addition, our group has also conducted various studies on the effects of TGF-β on hard tissue formation. For these reasons, we focused on TGF-β in this study as well.

Alkaline phosphatase (ALP) is known as the initial marker for the differentiation of mesenchymal cells into hard tissue-forming cells, such as osteoblasts or odontoblasts [21, 22]. We previously established an assessment tool for TGF-β activity by measuring ALP activity in HPDL cells [18]. It is also known that the *PAI-1* gene promoter has a TGF-β responsive region [23]. Therefore, the luciferase reporter-gene assay using a plasmid conjugated to the *PAI-1* promoter region has been widely used to investigate the regulation of transcription by TGF-β. We demonstrated that Hep-c fractionated from the G2 extract by heparin affinity chromatography enhanced ALP activity in HPDL cells and increased *PAI-1* gene promoter activity. These findings strongly suggest that an active form of TGF-β is present in Hep-c.

Acidic NCPs in bone have been associated with the gene expression and functional regulation of TGF-β [24–26]. Stains-all staining has been widely used for the detection of such acidic proteins, proteoglycans, nucleic acids and anionic polysaccharides [27–29]. We demonstrated that several Stains-all-positive NCPs with TGF-β activity coexisted in Hep-c isolated by heparin chromatography. Moreover, we were able to identify at least three NCPs, DMP1, MEPE, and BGN, in Hep-c. This finding led us to further investigate the interaction between these three NCPs and TGF-β.

The genes for most extracellular proteins that form bone, dentin, and enamel are known as the secretory calcium-binding phosphoprotein (SCPP) family [30–33]. Of the noncollagenous proteins in the SCPP family, the small integrin-binding ligand N-linked glycoprotein (SIBLING) family, composed of DMP1, MEPE, osteopontin, bone sialoprotein, and dentin sialophosphoprotein (DSPP), has common characteristics, such as a locus on chromosome 4 (4q21), similar exon structures, an arginine–glycine–aspartic acid motif that mediates cell attachment/signalling via integrin, and common conserved phosphorylation and *N*-glycan binding sites. We previously revealed that two DSPP-derived proteins, dentin sialoprotein and dentin phosphoprotein, are necessary for maintaining TGF-β1 activity in vitro [34]. In addition, several in vitro studies have revealed that small leucine-rich proteoglycans such as decorin and biglycan are able to bind to TGF-β and are candidates for the sequestration of TGF-β reservoirs [35–38]. In the present study, we demonstrated that approximately 11.3% of TGF-β activity remained when 1 µg of CF-hTGF-β1 alone was incubated and run on IE-HPLC. Moreover, we found that CF-hDMP1, CF-hMEPE, and CF-hBGN were able to rescue the loss of TGF-β1 activity, and this activity was increased by approximately 14.7–32.7% by binding to the three NCPs. Thus, our in vitro study suggests that these three NCPs are necessary for maintaining TGF-β1 activity. Considering that SIBLING family proteins have been associated with biomineralization as enhancers and/or inhibitors [39, 40], these proteins may also function in maintaining the activity of bioactive substances, such as TGF-β.

Our final goal was to develop a membrane that continuously releases osteoinductive factors that are resorbed in vivo and enables both quantitative and qualitative bone augmentation. We demonstrated that demineralized bone sheets with TGF-β activity enhance bone augmentation and that NCPs such as DMP1, MEPE and BGN in the demineralized sheet contribute the ability to retain bioactive molecules, such as TGF-β. Thus, the present study provides one of the necessary elements to develop the desired membrane. Further experiments, such as the measurement of membrane porosity and the measurement of sustained release time in vivo and in vitro*,* are required in the future. Moreover, it would be of tremendous interest to study the effectiveness of demineralized bone sheets with artificially added bioactive substances for bone augmentation around implants.

## Conclusions

Demineralized bone sheets are capable of inducing bone augmentation, and this ability is mainly due to TGF-β in the bone protein mixed with the sheets. The activity of TGF-β is maintained when binding to bone NCPs such as DMP1, MEPE, and BGN in the sheets. Demineralized bone sheets may be used for future implant treatments such as GBR or ridge preservation as membranes that are continuously able to release osteoinductive factors and enable quantitative and qualitative bone augmentation.

## Data Availability

The data are available from the corresponding author upon reasonable request.

## Abbreviations

ALP: Alkaline phosphatase
BGN: Biglycan
BMD: Bone mineral density
CF: Carrier free
DIA–MS: Data-independent acquisition–mass spectrometry
DMP1: Dentin matrix protein 1
DSPP: Dentin sialophosphoprotein
EDTA: Ethylenediaminetetraacetic acid
FDR: False discovery rate
GBR: Guided bone regeneration
HPDL: Human periodontal ligament fibroblasts
IE-HPLC: Ion exchange-high-performance liquid chromatography
MEPE: Matrix extracellular phosphoglycoprotein
mHAT9d: Enamel epithelial cell line
micro-CT: Microcomputed tomography
NCPs: Noncollagenous proteins
PAI-1: Plasminogen activator inhibitor-1
PBS: Phosphate-buffered saline
SCPP: Secretory calcium-binding phosphoprotein (SCPP)
SD: Sprague–Dawley
SDS–PAGE: Sodium dodecyl sulfate–polyacrylamide gel electrophoresis
SE: Standard error
SIBLING: Small integrin-binding ligand N-linked glycoprotein (SIBLING)
TGF-β: Transforming growth factor-beta
Tris: Tris(hydroxymethyl)aminomethane

## References

[1] Atwood DA (1971). Reduction of residual ridges: a major oral disease entity. J Prosthet Dent.

[2] Atwood DA (2001). Some clinical factors related to rate of resorption of residual ridges. J Prosthet Dent.

[3] Kalsi AS, Kalsi JS, Bassi S (2019). Alveolar ridge preservation: why, when and how. Br Dent J.

[4] Avila-Ortiz G, Chambrone L, Vignoletti F (2019). Effect of alveolar ridge preservation interventions following tooth extraction: a systematic review and meta-analysis. J Clin Periodontol.

[5] Stumbras A, Kuliesius P, Januzis G, Juodzbalys G (2019). Alveolar ridge preservation after tooth extraction using different bone graft materials and autologous platelet concentrates: a systematic review. J Oral Maxillofac Res.

[6] Khojasteh A, Soheilifar S, Mohajerani H, Nowzari H (2013). The effectiveness of barrier membranes on bone regeneration in localized bony defects: a systematic review. Int J Oral Maxillofac Implants.

[7] Lyu C, Shao Z, Zou D, Lu J (2020). Ridge alterations following socket preservation using a collagen membrane in dogs. Biomed Res Int.

[8] Lee JB, Chu S, Ben Amara H, Song HY, Son MJ, Lee J (2021). Effects of hyaluronic acid and deproteinized bovine bone mineral with 10% collagen for ridge preservation in compromised extraction sockets. J Periodontol.

[9] Stumbras A, Galindo-Moreno P, Januzis G, Juodzbalys G (2021). Three-dimensional analysis of dimensional changes after alveolar ridge preservation with bone substitutes or plasma rich in growth factors: randomized and controlled clinical trial. Clin Implant Dent Relat Res.

[10] Azangookhiavi H, Ghodsi S, Jalil F, Dadpour Y (2020). Comparison of the efficacy of platelet-rich fibrin and bone allograft for alveolar ridge preservation after tooth extraction: a clinical trial. Front Dent.

[11] Shirai M, Yamamoto R, Chiba T, Komatsu K, Shimoda S, Yamakoshi Y (2016). Bone augmentation around a dental implant using demineralized bone sheet containing biologically active substances. Dent Mater J.

[12] Urist MR (1965). Bone: formation by autoinduction. Science.

[13] Wozney JM, Rosen V, Byrne M, Celeste AJ, Moutsatsos I, Wang EA (1990). Growth factors influencing bone development. J Cell Sci Suppl.

[14] Hauschka PV, Chen TL, Mavrakos AE (1988). Polypeptide Growth Factors in Bone Matrix. Ciba Found Symp.

[15] Canalis E, McCarthy T, Centrella M (1988). Isolation of growth factors from adult bovine bone. Calcif Tissue Int.

[16] Seyedin SM, Thomas TC, Thompson AY, Rosen DM, Piez KA (1985). Purification and characterization of two cartilage-inducing factors from bovine demineralized bone. Proc Natl Acad Sci U S A.

[17] Harada K, Oida S, Sasaki S (1988). Chondrogenesis and osteogenesis of bone marrow-derived cells by bone-inductive factor. Bone.

[18] Nagano T, Oida S, Suzuki S, Iwata T, Yamakoshi Y, Ogata Y (2006). Porcine enamel protein fractions contain transforming growth factor-β1. J Periodontol.

[19] Tang SY, Alliston T (2013). Regulation of postnatal bone homeostasis by TGF-β. BoneKEy Rep.

[20] Tang Y, Wu X, Lei W, Pang L, Wan C, Shi Z (2009). TGF-β1-induced migration of bone mesenchymal stem cells couples bone resorption with formation. Nat Med.

[21] Sloan AJ, Rutherford RB, Smith AJ (2000). Stimulation of the rat dentine-pulp complex by bone morphogenetic protein-7 in vitro. Arch Oral Biol.

[22] Chen S, Gu TT, Sreenath T, Kulkarni AB, Karsenty G, MacDougall M (2002). Spatial expression of Cbfa1/Runx2 isoforms in teeth and characterization of binding sites in the DSPP gene. Connect Tissue Res.

[23] Hua X, Miller ZA, Wu G, Shi Y, Lodish HF (1999). Specificity in transforming growth factor β-induced transcription of the plasminogen activator inhibitor-1 gene: interactions of promoter DNA, transcription factor μE3, and Smad proteins. Proc Natl Acad Sci USA.

[24] Vetrone SA, Montecino-Rodriguez E, Kudryashova E, Kramerova I, Hoffman EP, Liu SD (2009). Osteopontin promotes fibrosis in dystrophic mouse muscle by modulating immune cell subsets and intramuscular TGF-β. J Clin Invest.

[25] Carvalheiro T, Malvar Fernández B, Ottria A, Giovannone B, Marut W, Reedquist KA (2020). Extracellular SPARC cooperates with TGF-β signalling to induce pro-fibrotic activation of systemic sclerosis patient dermal fibroblasts. Rheumatology.

[26] Kolb M, Margetts PJ, Sime PJ, Gauldie J (2001). Proteoglycans decorin and biglycan differentially modulate TGF-β-mediated fibrotic responses in the lung. Am J Physiol Lung Cell Mol Physiol.

[27] Green MR, Pastewka JV, Peacock AC (1973). Differential staining of phosphoproteins on polyacrylamide gels with a cationic carbocyanine dye. Anal Biochem.

[28] Myers JM, Veis A, Sabsay B, Wheeler AP (1996). A method for enhancing the sensitivity and stability of stains-all for phosphoproteins separated in sodium dodecyl sulfate-polyacrylamide gels. Anal Biochem.

[29] Goldberg HA, Warner KJ (1997). The staining of acidic proteins on polyacrylamide gels: enhanced sensitivity and stability of "stains-all" staining in combination with silver nitrate. Anal Biochem.

[30] Kawasaki K, Weiss KM (2006). Evolutionary Genetics of Vertebrate Tissue mineralization: the origin and evolution of the secretory calcium-binding phosphoprotein family. J Exp Zoo B Mol Dev Evol.

[31] Kawasaki K, Buchanan AV, Weiss KM (2007). Gene duplication and the evolution of vertebrate skeletal mineralization. Cells Tissues Organs.

[32] Kawasaki K (2011). The SCPP Gene Family and the Complexity of Hard Tissues in Vertebrates. Cells Tissues Organs.

[33] Rowe PS (2012). The chicken or the egg: PHEX, FGF23 and SIBLINGs unscrambled. Cell Biochem Funct.

[34] Yamakoshi Y, Kinoshita S, Izuhara L, Karakida T, Fukae M, Oida S (2014). DPP and DSP are necessary for maintaining TGF-β1 activity in dentin. J Dent Res.

[35] Hildebrand A, Romaris M, Rasmussen LM, Heinegard D, Twardzik DR, Border WA (1994). Interaction of the small interstitial proteoglycans biglycan, decorin and fibromodulin with transforming growth factor β. Biochem J.

[36] Iozzo RV, Murdoch AD (1996). Proteoglycans of the extracellular environment: clues from the gene and protein side offer novel perspectives in molecular diversity and function. FASEB J.

[37] Hyytiainen M, Penttinen C, Keski-Oja J (2004). Latent TGF-β Binding Proteins: Extracellular Matrix Association and Roles in TGF-β Activation. Crit Rev Clin Lab Sci.

[38] Baker SM, Sugars RV, Wendel M, Smith AJ, Waddington RJ, Cooper PR (2009). TGF-β/extracellular matrix interactions in dentin matrix: a role in regulating sequestration and protection of bioactivity. Calcif Tissue Int.

[39] Huang B, Sun Y, Maciejewska I, Qin D, Peng T, McIntyre B (2008). Distribution of SIBLING proteins in the organic and inorganic phases of rat dentin and bone. Eur J Oral Sci.

[40] Qin C, Baba O, Butler WT (2004). Post-translational modifications of sibling proteins and their roles in osteogenesis and dentinogenesis. Crit Rev Oral Biol Med.

